# Sociological and religious interpretations of Adinkra symbols: qualitative analysis of the 2025 inaugural outfit of Ghana's president

**DOI:** 10.3389/fsoc.2025.1642863

**Published:** 2025-11-17

**Authors:** Ronald Osei Mensah, Anthony Bordoh, Andrews Acquah, Eric Bruce-Amartey Jnr, Papa Kofi Nunoo

**Affiliations:** 1 Faculty of Media Technology and Liberal Studies, Social Development Department, Takoradi Technical University, Takoradi, Ghana; 2 Department of Social Studies Education, University of Education, Winneba, Ghana; 3 National Centre for Research into Basic Education, University of Education, Winneba, Ghana; 4 Textile Design and Technology Department, Faculty of Applied Arts and Technology, Takoradi Technical University, Takoradi, Ghana; 5 Political Science and History Department, University of Ghana, Accra, Ghana

**Keywords:** Adinkra symbols, political symbolism, Ghanaian culture, inaugural attire, symbolic interactionism, semiotic theory

## Abstract

**Introduction:**

Adinkra symbols, deeply embedded in Ghanaian culture, serve as potent non-verbal communicative tools that convey philosophical, religious, and sociopolitical meanings.

**Methods:**

This study explores the sociological, religious, and fashion interpretations of the Adinkra symbols incorporated into the 2025 inaugural outfit of Ghana's President. Anchored in a qualitative research design and aligned with a constructivist research philosophical position, the study draws on insights from 28 purposively selected participants, including traditional leaders, fashion designers, museum curators, and cultural preservatists.

**Results and discussion:**

The findings reveal that “Sankofa”, “Gye Nyame”, “Nyame Dua”, and “Dwennimmen”, key symbols present in the attire, collectively signify governance introspection, divine authority, ethical leadership, and humility in power. Through the lens of Symbolic Interactionism and the Semiotic Theory of Fashion, the study demonstrates how these symbols serve as ideological markers, reinforcing national identity, political philosophy, and ethical governance. The results highlight the critical role of indigenous symbolism in shaping public perceptions of leadership, fostering national unity, and maintaining cultural continuity in Ghana's political landscape. The study calls for the integration of traditional symbols into governance, education, and leadership training to strengthen national identity and ethical governance.

## 1 Introduction

Adinkra symbols, initially created by the Asante and embedded in broader Akan-speaking traditions, represent a sophisticated visual system that communicates proverbs, values, and philosophical reflections, particularly in funerary and ceremonial contexts (Owusu, 2019; Arthur, 2017; Willis, 1998; Kuwornu-Adjaottor et al., 2016). Once regionally confined, these ideograms have now become national cultural markers, visible on textiles, buildings, official emblems, and everyday objects (Asiedu, 2022). Political leaders often appropriate Adinkra to signal cultural alignment or national unity. For example, President John Mahama, an ethnic Gonja, wore kente cloth featuring Adinkra motifs during his inauguration, an act interpreted variously as symbolic, inclusive, or politically calculated (Yankah, 1995).

These symbols carry multivocal meanings and are expressed through a range of media, including gestures, oral language, and visual representation (Owusu, 2019). Among Ghanaian youth, they shape cultural identity by offering moral guidance, affirming cultural heritage, and encouraging respect for elders and communal responsibility (Blount and Brookins, 2022). They also encode emotional messages that support social cohesion, promote non-violent interaction, and nurture psychological resilience during adversity (Dzokoto et al., 2018; Owusu, 2019). More than ornamental designs, Adinkra serve pedagogical, interpersonal, and emotional functions within Akan society (Owusu, 2019; Dzokoto et al., 2018), while also providing African-American communities with symbolic links to ancestral African traditions (Dzokoto et al., 2018).

Adinkra is not a fixed or universally agreed-upon system; rather, its meanings can be ambiguous, fluid, and even contested across Ghana's multi-ethnic and politically complex landscape. As such, Adinkra functions not merely as decorative art but as a deeply symbolic, and at times, contentious medium through which moral, historical, and political narratives are expressed, negotiated, and reinterpreted (Akou, 2004; Mato, 2001; Owusu, 2019; Dzramedo et al., 2013; Bouska and Beatty, 1978).

This symbolic potency is particularly evident in the realm of political leadership, where strategic sartorial choices often serve to affirm cultural identity and transmit subtle yet powerful political messages. The presidential inauguration, a ritualized performance of political continuity and transformation, is saturated with meaning. In Ghana, the use of Adinkra symbols in presidential attire has gained traction as a means of reinforcing national identity, signaling ideological intentions, and connecting modern governance to ancestral wisdom. The symbols used on such occasions do not merely embellish clothing; they articulate values, project power, and invite public interpretation. As Martino (2018) and Aflakpui et al. (2023) argue, Adinkra symbols are pedagogical tools, rich with multivalent meanings and capable of encoding both timeless philosophies and contemporary concerns. Aboagyewaa-Ntiri et al. (2018) even locate these symbols within the framework of Maslow's hierarchy of needs, asserting their relevance to fundamental human aspirations, while Danzy (2009) positions them as a system of ideographic writing reflecting the beliefs and identities of a people.

Historically, Ghana's encounter with colonialism disrupted indigenous dress practices by introducing Western fashion as a marker of modernity and elite status. Westernized education and administrative systems led to the internalization of European dress codes, especially among the Ghanaian elite. As Essel (2013, p. 19) notes, this colonial legacy prompted Ghana's first president, Kwame Nkrumah, to champion a cultural and sartorial decolonisation project, advocating a “national dress” that reclaimed indigenous identity and rejected Eurocentric norms. This was not just a domestic gesture; at international diplomatic forums like the United Nations in the 1950s, Ghanaian and Nigerian delegates deliberately wore national attire in defiance of the Western suit, presenting African dress as a symbol of sovereignty and authenticity (Rogers et al., 2013; Akou, 2004).

While existing scholarship has examined the philosophical, historical, and communicative dimensions of Adinkra symbolism (Kissi et al., 2019; Owusu, 2019; Aboagyewaa-Ntiri et al., 2018), relatively little attention has been paid to how these symbols function within contemporary political settings, particularly presidential inaugurations. More specifically, there remains a gap in understanding how Adinkra symbols are religiously and sociologically interpreted in such contexts, and how they contribute to the symbolic construction of political identity. Additionally, few studies have analyzed these symbols through the lens of symbolic interactionism, a theory that foregrounds the role of symbols in shaping social meaning and interaction.

This study, therefore, aims to explore the Adinkra symbols featured on the 2025 inaugural outfit of Ghana's President, applying symbolic interactionism to understand how these symbols communicate political, religious, and cultural meanings. It investigates how the selection, placement, and interpretation of these symbols by various societal actors contribute to constructing political identity, shaping public perception, and influencing broader socio-political dynamics in Ghana. By doing so, the research seeks to bridge a critical gap in the literature, offering new insights into the intersection of symbolism, politics, and culture.

The following research questions guide the study:

a) How are the Adinkra symbols on the President's inaugural outfit interpreted religiously?
b) What are the sociological interpretations of the Adinkra symbols on the President's inaugural outfit?
c) What is the societal significance and implications of the Adinkra symbols displayed on the President's inaugural outfit?

## 2 Theoretical review

### 2.1 . Symbolic interactionism

Symbolic interactionism, a prominent sociological framework, delves into the intricate processes through which individuals construct meaning through their interactions with others and the surrounding world. This perspective underscores the pivotal role of symbols, language, and interpretation in shaping social reality. Drawing upon key scholarly works of (Mardon et al., 2021; Husin et al., 2021; Kotarba, 2014; Brooks-Yip, 2023; Stryker and Annual Reviews, 2008; Carter and Fuller, 2016, 2015), this review explores the core tenets of symbolic interactionism and discusses its relevance and application to analyzing the Adinkra symbols adorning the 2025 inaugural outfit of Ghana's President. Meaning is a social construct that arises from interaction, according to symbolic interactionism, rather than an intrinsic quality of things or behaviors. According to Blumer (Mardon et al., 2021), three basic principles underlie human behavior: people act according to the meanings that objects contain for them; these meanings are derived via social interaction; and meanings are constantly negotiated and altered through the process of interpretation. This emphasizes how shared symbols promote understanding and communication, underscoring the dynamic and ever-changing character of meaning-making. Further highlighting the importance of symbols as a communication tool, especially at the individual level where complex interpretations influence interpersonal dynamics, are Mardon et al. (2021). In further detail, Carter and Fuller (2016) describe how people classify and characterize things and circumstances according to their subjective meanings, influenced by their unique experiences and social contexts. This interpretive process allows for multiple layers of meaning to be embedded within a single symbol, enriching its communicative potential. Stryker and Annual Reviews (2008) discusses Mead's contributions, which form the basis of symbolic interactionism. Mead's focus on the social process as the primary factor forming society, the self, and social interaction—each of which in turn influences the others—is emphasized by Stryker and Annual Reviews (2008). Understanding how people create their identities and negotiate the intricacies of the social environment depends on this connectivity between the self and society. According to Mead, social contact and internalizing the viewpoints of others are how the “self” emerges. This practice of “taking the role of the other” enables people to predict other people's reactions and modify their actions accordingly, which aids in the continuous meaning negotiation. The Ghanaian President's inaugural attire, Adinkra symbols, can be examined via the particularly illuminating lens of symbolic interactionism. These symbols have deep cultural and historical significance and stand for intricate concepts and ideals, making them more than just ornamentation. Understanding the meaning of these symbols in Ghanaian society and the larger cultural context can be obtained by looking at how people perceive and engage with them. Meaning-making has a significant impact on identity construction and expression, as discussed by Brooks-Yip (2023). This is particularly pertinent to comprehending the President's clothing choice and its symbolic meaning. One may argue that the choice and presentation of Adinkra symbols is a conscious act of communication that sends particular signals to audiences both at home and abroad. Analyzing the meanings that different people and groups assign to the Adinkra symbols is necessary to apply symbolic interactionism to this subject. This necessitates taking into account both the individual interpretations of individuals who are viewing the symbols as well as the social and cultural environment in which they are presented. In applied social research, Kotarba (2014) highlights the value of translational science research and promotes an emphasis on closing the gap between theory and practice. In this case, it refers to comprehending how real-world occurrences, like the symbolic communication the President's clothing conveys, may be effectively analyzed using the theoretical framework of symbolic interactionism. A thorough literature analysis of recent studies on symbolic interactionism is presented by Husin et al. (2021), providing additional insights into its use across a variety of contexts. Their work underscores the versatility of this framework in examining a wide range of social phenomena, from interpersonal interactions to large-scale cultural trends. A helpful framework for understanding how meaning is socially constructed and how it significantly affects people's behavior and identities is provided by symbolic interactionism. This theory can be used to comprehend better the cultural significance of Adinkra symbols and the many signals they communicate to various audiences by analyzing them on the Ghanaian President's inaugural attire. Through symbolic interactionism, these symbols can be studied to shed light on the complex ways that cultural legacy is communicated, negotiated, and passed down.

### 2.2 Semiotic theory of fashion

With its foundations in linguistics, semiotic theory offers a robust framework for understanding fashion as a complex communication system. This review engages the foundational ideas of semiotics. It examines their relevance and applicability to the analysis of fashion, particularly in traditional and political contexts, drawing on works such as Mikerina (2016), Boero (2023), Honeycutt (2021), Karunaratne (2018), Khrystych (2019), and Inayatullah and Wahjudi (2023), along with Vincent (2022) and Mikerina (2016).

Roland Barthes's *The Fashion System*, as cited in Karunaratne (2018), revolutionized the study of fashion by interpreting clothing as a language comprising signifiers (garments) and signifiers (their meanings), shaped by culture. This framework shifts the analytical lens from the utilitarian or aesthetic attributes of fashion to its symbolic and communicative dimensions. Boero (2023) furthers this by emphasizing the axiological function of fashion, positioning it as a mechanism for transmitting societal values. She distinguishes between “custom”, which reflects dominant norms, and “clothing,” which serves as a medium for personal expression. This distinction is essential for understanding the dynamic between conformity and individuality in fashion expression, including in political contexts.

Barthes's framework introduced the idea of semiotic codes, culturally embedded systems that structure the interpretation of fashion signs. Honeycutt (2021), for example, illustrates the application of Barthes's five semiotic codes through the analysis of costume design in French cinema, showing how even minor stylistic choices contribute to an overall narrative. This analytical precision can be fruitfully applied to Ghanaian presidential attire, especially the 2025 inauguration, where Adinkra symbols serve not merely as decoration but as conveyors of layered political, cultural, and spiritual messages. Each symbol's selection, placement, and chromatic coordination encodes meanings that reflect Ghanaian values and identity.

However, while Barthes's theory offers a compelling lens, its application in non-Western, traditional, and political settings such as Ghana requires thoughtful adaptation. As Mikerina (2016) argues, the theory's original emphasis on representation may not fully capture the material and experiential aspects of contemporary or indigenous fashion systems. In the context of Ghana's presidential regalia, where cloth is embedded with sacred and historical meaning, Barthes's focus on abstraction and symbolism may overlook lived, embodied practices and ritualistic dimensions. Indeed, the tactile experience of wearing Adinkra cloth, its handcrafted production, and its invocation of ancestral knowledge suggest that meaning arises not only from symbolic systems but also from socio-historical context and corporeal engagement.

Furthermore, the application of semiotic theory in political dress risks imposing rigid interpretations on fluid cultural symbols. The presidential attire, though rich in signification, operates within a space that is not purely aesthetic or symbolic, but also performative and strategic. Inayatullah and Wahjudi (2023), through their Barthesian analysis of urban fashion, provide a methodological framework that can be adapted to this context. However, they acknowledge the hybrid and often contradictory meanings produced in public fashion performances. Vincent (2022) offers an instructive example in her semiotic analysis of *Vogue* magazine covers, emphasizing the socio-political embeddedness of fashion imagery. Similarly, Khrystych (2019) and Karunaratne (2018) highlight how fashion functions within power structures, ideologies, and cultural negotiations.

Therefore, while semiotic theory remains an insightful tool, its application to traditional political dress, like the Ghanaian president's attire, must be critically calibrated. The theory must be extended to consider not only the symbolic meanings of signs but also their ritual function, historical continuity, and political intentionality. By doing so, the study moves beyond a Western-centered fashion semiotics and toward a more contextualized understanding of how traditional symbols like Adinkra communicate authority, cultural heritage, and national identity.

**Figure 1:**
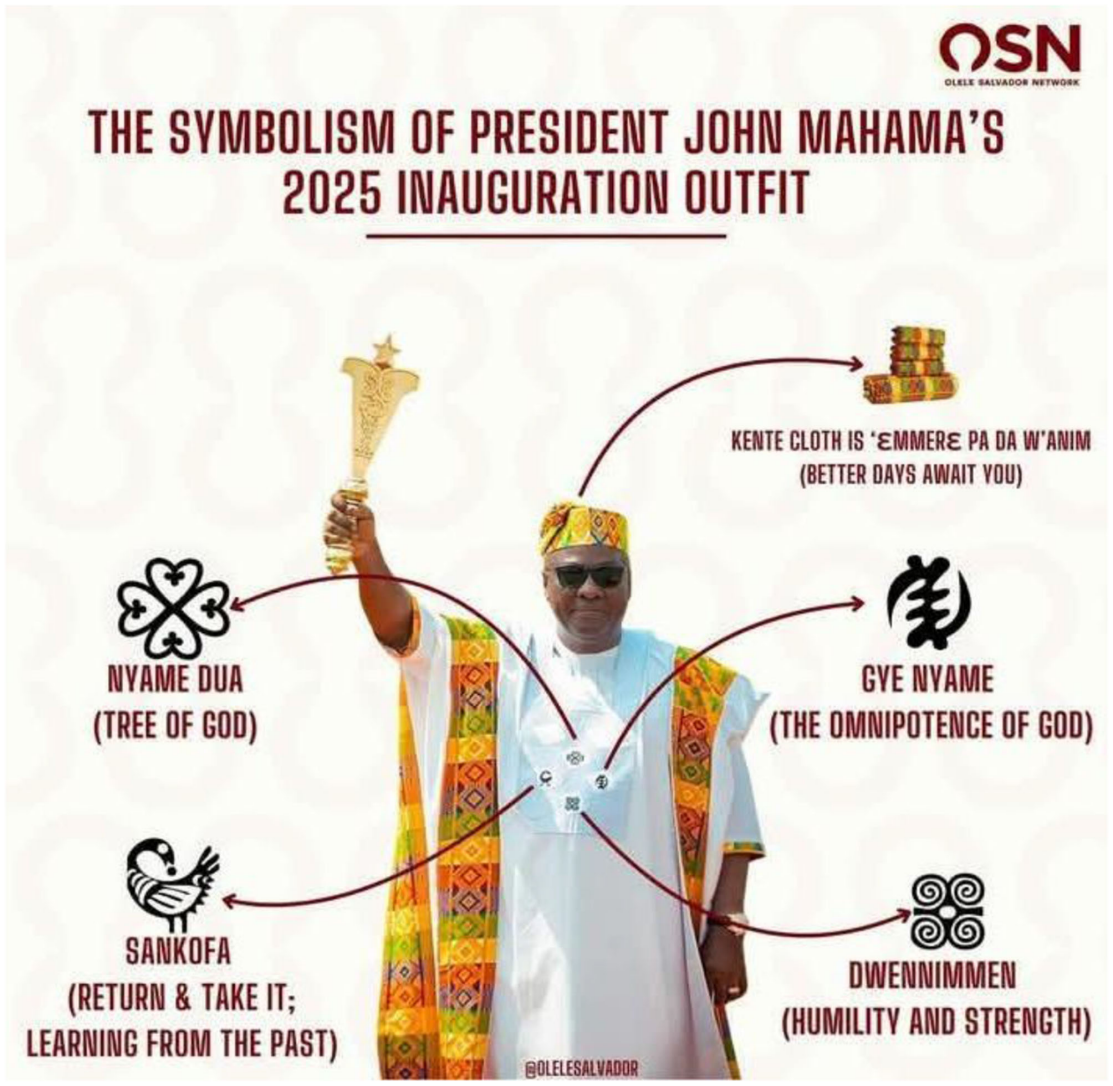
Key symbolic perspectives of the President's inaugural outfit. Source: Okine Isaac (Ghana web, 2025). Reproduced with permission from @olelesalvador.

In sum, the semiotic approach helps decode the intricate symbolism in presidential fashion, but it requires modifications to accommodate the embodied, ritualistic, and political dimensions of traditional African dress. Figure 1 below visualizes this adapted theoretical interpretation of the President's attire within a semiotic framework.

### 2.3 Empirical review

#### 2.3.1 Religious interpretations of traditional symbols of outfit

In many different cultures, traditional symbols embroidered into garments have deep religious meaning. These symbols serve as a visual language that conveys intricate spiritual ties, values, and beliefs. This review delves into the religious interpretations of such symbols, drawing upon a range of scholarly works to provide a nuanced understanding of their multifaceted nature. According to Sunday et al. (2024), symbols are essential to religious experience because they allow for communion with the divine and serve as concrete representations of abstract ideas. They provide a way to unite people to a common spiritual concept and to articulate the inexpressible. African symbols are inherently religious, according to Anyigor and Olekaibe (2022), who also stress how they relate to the interaction between the sacred and the profane in African cosmology. These symbols are more than just ornaments; they represent profound religious meanings that must be carefully interpreted. These meanings are frequently transmitted through the generations and woven into cultural tales. Further highlighting the religious role of apparel and textiles, Akinbileje (2014) mentions their use in rites and rituals as well as their storage of supernatural abilities, giving them spiritual value and serving as a conduit between the physical and spiritual realms. Traditional symbols in religion are frequently entwined with how cultural identity is constructed and expressed. Mawuli (2019) investigates how Adinkra iconography on Ghanaian traditional attire is used to transmit and embody history. These emblems, which are passed down via family and custom, are potent representations of spiritual and cultural identification that bind people to their ancestors and strengthen a feeling of common identity. In their discussion of the iconography and iconology of Adinkra symbols, Aflakpui et al. (2023) highlight the significance of these symbols in communicating abstract truths, cultural values, and complicated information. By serving as a visual representation of shared values and customs, the employment of these symbols in apparel strengthens cultural identification. It acts as a potent message of solidarity and inclusion within a particular cultural group. This visual language allows for the communication of shared values and beliefs, strengthening community bonds and reinforcing cultural heritage. Amoateng (2018) explores the symbolic theology inherent in Adinkra symbols, arguing that they serve as a means of passing along religion from one generation to the next. The author illustrates the deep religious metaphors expressed by these symbols with the examples of “Gye Nyame” (except God) and “Owuo Atwee Nyame” (Death has no power over God). These symbols convey notions about the character of God, the relationship between humans and the divine, and the victory of good over evil, all of which are intricate theological concepts. The symbolic designs of African textile art are further examined by Labode and Braide (2022), who emphasize the elaborate patterns and motifs that have religious significance. These designs often reflect cosmological beliefs, spiritual narratives, and the relationship between humanity and the divine, offering a visual representation of the spiritual world and its connection to human experience. The intricate details and symbolism woven into these fabrics serve as a constant reminder of the spiritual dimension of life. While traditional symbols hold deep religious significance, their meanings can be misinterpreted or misused in contemporary society. Anyigor and Olekaibe (2022) caution against the mislabeling of African religious treasures as mere pagan idols, emphasizing the need to appreciate their true meaning and the rich cultural heritage they represent. As these symbols become increasingly commodified and globalized, it is crucial to maintain their original meaning and prevent their appropriation for commercial or superficial purposes.

#### 2.3.2 Sociological interpretations of Adinkra and traditional symbols

Originating in Ghana's Asante culture, Adinkra symbols have evolved beyond their original cultural setting to become important representations of wider Ghanaian and even pan-African customs, beliefs, and values (Aflakpui et al., 2023; Deguara and Nutbrown, 2018). According to Aflakpui et al. (2023), these symbols, which are frequently found on textiles, ceramics, and other artifacts (Deguara and Nutbrown, 2018), convey intricate concepts and beliefs through visual representations. Their interpretations, which have their roots in social contexts, provide important insights into the societal structures and cultural dynamics of the Akan people as well as other groups. Adinkra patterns' elaborate patterns and symbolic meanings reveal a deep comprehension of morality, social order, and the interdependence of all life. Their capacity to communicate nonverbally is a crucial component of Adinkra's sociological relevance, according to Aflakpui et al. (2023). Adinkra symbols are a potent means of communicating ideas about human conduct, attitudes, and even the universe's order in a civilization where symbolic expression is essential (Aflakpui et al., 2023). According to Aflakpui et al. (2023), this non-verbal communication encompasses cultural values and proverbial meanings in addition to aesthetics. For example, the use of Adinkra symbols in apparel can represent underlying social tensions and dynamics within families and communities by expressing social commentary, such as co-wife competition (Deguara and Nutbrown, 2018). This subtle mode of communication enables the expression of complicated thoughts and feelings within a framework understood by the culture. The symbols act as a shared language, facilitating communication and understanding within the community. According to Aflakpui et al. (2023), Adinkra symbols also play a significant role in the development and comprehension of cultural identity. They serve as visual indicators that assist people in defining their community's expectations, values, and culture (Aflakpui et al., 2023). The use of Adinkra symbols in national emblems and currencies shows that this role in forming identity also applies to national identity (Aflakpui et al., 2023; Ankora, 2022). Social cohesiveness and cultural continuity are enhanced by the employment of these symbols, which strengthen a feeling of shared heritage and collective identity. The preservation of social harmony and the development of a feeling of communal belonging depend heavily on this sense of shared identity. The symbols serve as a unifying factor, tying people to their cultural roots and enhancing their sense of place within society. Beyond their cultural and national significance, Adinkra symbols also hold relevance for environmental sustainability education (Adom et al., 2018). The Sankofa symbol, for example, encourages reflection on past practices and the adoption of sustainable lifestyles from ancestors Adom et al. (2018). This connection between cultural symbols and environmental awareness highlights the potential of Adinkra to promote responsible environmental practices and intergenerational equity. By drawing on traditional wisdom and cultural values, Adinkra symbols can inspire a renewed commitment to environmental stewardship and sustainable living. Moreover, Adinkra symbols have found application in various fields, including interior decoration (Ofori et al., 2023) and product advertisement (Oladumiye, 2018). Their use in interior design reflects a growing interest in incorporating indigenous cultural elements into contemporary aesthetics (Ofori et al., 2023). This integration of traditional symbols into modern design creates a unique visual language that celebrates cultural heritage while embracing contemporary trends. In advertising, Adinkra symbols can enhance the cultural relevance and appeal of products, particularly in the African context (Oladumiye, 2018). This demonstrates the versatility and adaptability of Adinkra symbols in diverse contexts. Their ability to transcend traditional boundaries and find relevance in modern applications speaks to their enduring power and cultural significance. Their social interpretations are further complicated by the philosophical foundations of Adinkra symbols, such as the idea of “Nyàmé ϕwú Nà Màwù” (Mensah et al., 2020), which examines the duality of existence and the interconnection of life and death. These philosophical ideas offer insights into the Akan people's worldview and ethical system since they frequently mirror larger societal values and beliefs. Examining these intellectual foundations demonstrates the richness and depth of Adinkra symbolism, emphasizing its function in forming moral principles and directing moral conduct in the community. The poetic interpretations of Adinkra symbols, as discussed by Ansong (2022), provide an insightful lens through which to understand their meanings and cultural importance. These interpretations distill the core messages of the symbols into a literary format, thereby broadening their accessibility and aiding in the preservation of cultural heritage. The incorporation of poetic language introduces an additional dimension of artistic expression to the understanding of Adinkra, amplifying their aesthetic charm and emotional impact. Analyzing Adinkra from diverse perspectives—sociological, philosophical, and artistic—deepens our comprehension of these symbols and their persistent significance in modern society. Furthermore, the application of Adinkra in textiles, particularly concerning national identity, Ankora (2022), highlights their role as vital cultural markers and representations of collective identity. The ongoing utilization and adaptation of Adinkra symbols within contemporary Ghanaian culture illustrate their crucial role in shaping cultural identity, fostering social unity, and passing down traditional values and beliefs to future generations.

#### 2.3.3 Relevance of traditional symbols in African traditional societies

Traditional symbols are integral to the fabric of African societies, serving as powerful vehicles for cultural transmission, social cohesion, and spiritual expression. These symbols, woven into the tapestry of daily life and rituals, convey intricate meanings and values that shape individual identities and collective experiences. They act as a bridge between the past and the present, connecting communities to their ancestral heritage and providing a framework for understanding the world. From intricate patterns adorning clothing and pottery to symbolic gestures and rituals, these visual and performative expressions embody a rich cultural vocabulary that speaks to the heart of African traditions. Numerous researchers have investigated the complex functions of symbols within African cultures. Udechukwu (2019) delves into the importance and application of cultural symbols in modern African contexts, explicitly focusing on Igbo symbols as a case study. Her research classifies various categories of symbols, including those related to animals, rituals, royalty, ancestry, and culture, emphasizing their varied roles in upholding social cohesion, articulating beliefs, and fostering connections with ancestral legacies. For instance, Udechukwu (2019) highlights the symbolism of thrones as representations of power and the cultural necessity of honoring elders, who serve as links to ancestral wisdom. This respect for ancestral figures illustrates the critical role of lineage and tradition in Igbo society, where the insights and guidance of previous generations are deeply esteemed. Additionally, the employment of animal symbols, such as the leopard denoting strength and the tortoise signifying wisdom, further enhances the rich symbolic lexicon of the Igbo community. The effectiveness of cultural symbols in product advertising is examined by Oladumiye (2018), who highlights the significance of comprehending cultural affinities while utilizing symbols in communication. His research highlights the significance of specific African cultural icons, including cocoa, kola nuts, and cowries, indicating their capacity to connect with target audiences. This study demonstrates the usefulness of cultural symbols in modern situations, demonstrating their continued significance outside of conventional contexts. Advertisers can develop more successful ads that engage customers more deeply by knowing the cultural meaning of these symbols.

**Figure 2:**
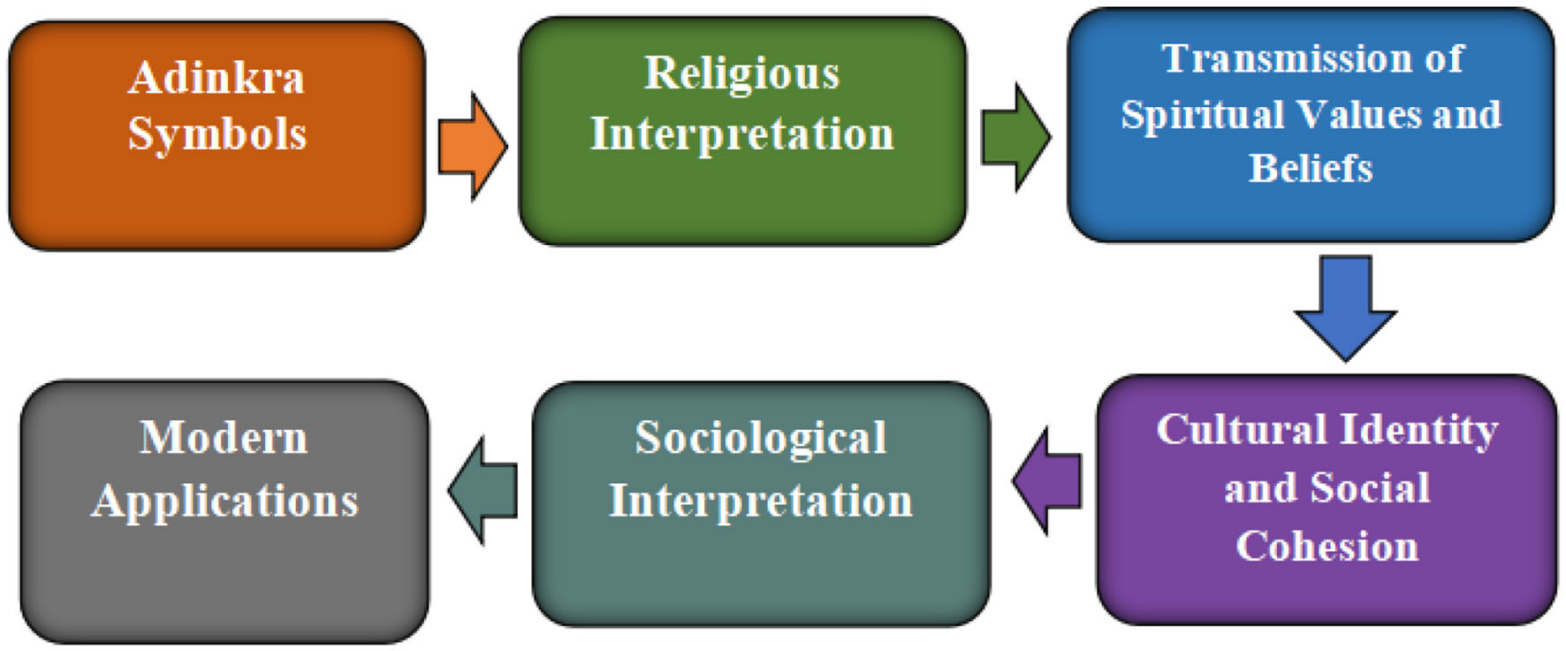
A Conceptual framework for understanding the role of traditional symbols in society. Source: Authors' construct (2025).

Using a phenomenological perspective, Anyigor and Olekaibe (2022) explore the meanings of symbols and symbolism in African Traditional Religion. They contend that symbols act as mediators, expressing the complete presence and significance of things and occurrences, and that they receive their meaning from social consensus. The writers examine certain symbols, such as cowries and white chalk, and explain how they represent wealth, chastity, and purity. This examination highlights the spiritual aspect of symbols, demonstrating their ability to strengthen religious convictions and establish a person's connection to the divine. By establishing a concrete connection between the material and spiritual worlds, the incorporation of symbols into rituals and rites enhances their spiritual power. Ansong (2022) explores Adinkra symbols, a set of ideographs indigenous to the Akan people of West Africa, focusing on their artistic, gendered, and poetic dimensions. The study highlights the role of Adinkra symbols in expressing cultural identity and preserving historical memory. Ansong's work emphasizes the dynamic nature of symbols, demonstrating their adaptability to various forms of expression, including poetry and visual art. This perspective underscores the creative potential of symbols and their capacity to evolve alongside cultural practices. The intricate designs and symbolic meanings embedded within Adinkra cloth serve as a testament to the artistic ingenuity and cultural depth of the Akan people. Adinkra symbols are examined by Jecty (2022) as a way to instill in the next generation beneficial Ghanaian personality traits. This study highlights the moral and pedagogical significance of traditional symbols, underscoring their role in fostering desirable character traits and imparting cultural values. The significance of cultural continuity and the function of symbols in passing down legacy and influencing future generations are emphasized by Jecty's work. Communities can guarantee the preservation of their cultural heritage and give the next generation a sense of pride and belonging by teaching kids the meanings and values connected to these symbols. All of these studies show how traditional symbols are widely used in African societies. These symbols continue to be effective means of communication, identity construction, and cultural preservation, as seen by their use in modern media and education, as well as their importance in religious rituals and social systems. They serve as an essential conduit to the past, a source of inspiration in the present, and a roadmap for the future, guaranteeing the survival of African customs and the timeless potency of symbolic expression. Figure 2 depicts the role of traditional symbols in society.

### 2.4 Conceptual framework

The intricate relationship between culture, religion, and society can be observed through the lens of symbolic representation. Cultural symbols (Adinkra Symbols) are imbued with religious interpretations, conveying their spiritual significance. This religious interpretation plays a crucial role in transmitting spiritual values and beliefs across generations, fostering a strong sense of cultural identity and social cohesion within a community. The shared cultural identity, in turn, leads to sociological interpretations of these symbols, reflecting the prevailing social norms and behaviors. These sociological interpretations find contemporary applications in various fields, such as fashion, further reinforcing their cultural significance. The continued relevance and utilization of these symbols in modern contexts contribute significantly to the formation of national identity, unifying people under a shared framework of cultural and symbolic heritage.

## 3 Methodology

### 3.1 Research approach and design

This study adopted a qualitative research approach, consistent with a constructivist philosophical orientation. The goal was to explore the sociological, religious, and fashion-related interpretations of the 2025 inaugural outfit of Ghana's President. Given the symbolic and cultural dimensions of the topic, qualitative research was appropriate for uncovering the subjective meanings and values attached to clothing within the Ghanaian context. The study relied on qualitative interviews to gather insights. Semi-structured interviews were conducted with individuals knowledgeable about Ghanaian fashion, symbolism, religion, and sociopolitical culture. This method allowed for flexibility in exploring participants' views while maintaining a consistent focus on the research objectives. Qualitative interviews were particularly suitable for understanding how individuals interpret and assign meaning to symbols, attire, and public representation. As Van Burg et al. (2022) note, qualitative interviewing is a powerful tool for investigating complex social phenomena and capturing rich, context-specific perspectives.

### 3.2 Participants

Fashion designers, Queens and Chiefs of traditional societies, traditional leaders and elders of Ghanaian society, Museum curators, and cultural preservatists were the participants in the study. These people possess unique insights into the symbolism and cultural significance of clothing within their respective groups. Their experiences and interpretations of clothing are essential to uncovering the depth of meaning and the role clothing plays in their lives. Their observations and reactions to individuals based on their clothing choices offered a more comprehensive view of how society responds to clothing symbolism. We spent extended periods within the communities we studied, gaining their trust and interacting with them. This helped us understand how clothing is used as a symbol and its sociological, religious and fashion significance within Ghanaian society.

Primary data was utilized in this study. A semi-structured interview guide was used as a data collection tool. Semi-structured interviews, through open-ended questions and active listening, allowed participants to share their insights, experiences, and opinions about clothing symbolism within the Ghanaian cultural system. This approach facilitated the exploration of diverse viewpoints while ensuring a focused discussion on the study's objectives. The exploratory research design was advanced to examine the dynamics of clothing symbolism activities (Ennin and Mensah, 2022). Participants were selected based on their knowledge and experience as clothing enthusiasts. They have firsthand knowledge about the symbolism behind certain types of clothing within their respective societies. Not only do they have firsthand knowledge, but some of these participants exposed what it means to wear certain attire in signaling status, affiliation, or involvement in an activity and the community at large (Mensah and Korankye, 2025).

A total of 28 participants were purposefully sampled for this study, including 8 Adinkra fashion designers and artisans, seven general fashion designers and enthusiasts, 6 Museum curators and cultural preservers, and 7 Traditional leaders and elders in the Ghanaian society. Purposive sampling was chosen for this study due to its alignment with the research objectives and the specific expertise required from the participants. According to Saunders (2014), purposive sampling allows researchers to intentionally select participants who possess firsthand knowledge and experience related to the research topic. The choice of a sample size of 28 participants in Ghana was influenced by considerations of feasibility, generalizability to the Ghanaian context, depth of analysis, and the desire to achieve data saturation. Data saturation occurs when no new themes or insights emerge from additional interviews, indicating that a comprehensive understanding of the research topic has been reached (Fusch and Ness, 2015).

The participants were selected based on their knowledge and experience related to clothing symbolism within the Ghanaian cultural and traditional system. This selection criterion ensured that the participants had the relevant expertise to contribute meaningfully to the study's objectives. The Ashanti Region of Ghana and the Volta Region of Ghana were used as places for the collection of data from participants. These regions were purposively selected because of their understanding and rich insights into the Ghanaian cultural and traditional system, and as originators of most of the traditional clothing we wear as Ghanaians. These regions are considered the cultural heartbeat of Ghana (Dennis, 2018; Skinner, 2013). However, we acknowledge that this regional focus may introduce a degree of bias, as other areas in Ghana, such as the Northern Regions, Greater Accra, and Zongo communities, also possess rich cultural and sartorial traditions that could offer alternative symbolic interpretations. While these regions are often recognized for their centrality in Ghana's textile heritage, the characterization of them as the “cultural heartbeat of Ghana” reflects a specific cultural perspective rather than a universally accepted view. As such, this study's findings should be understood within the context of this regional lens, and future research would benefit from a more geographically diverse sample to capture the full spectrum of Ghanaian cultural symbolism. Participants were identified through local community organizations in Manhyia (Kumasi), Bantama (Kumasi), Ejisu (Kumasi), Hohoe (Volta), Keta (Volta) and Anlo (Volta).

The interviews were conducted in person, and some were conducted through phone communication methods. The interview process for this study on clothing as a symbol in Ghana, particularly the President's inaugural cloth, was conducted using a semi-structured approach, allowing for flexibility while ensuring key topics were explored consistently across participants. Interviews were conducted individually with participants from various backgrounds. The duration of each interview varied depending on participant availability and the depth of discussion, typically lasting between 30 min to an hour. Interviews took place in settings conducive to open and candid conversations, including community centers, workplaces and neutral public locations chosen to accommodate participants' preferences and ensure privacy. Questions were designed to elicit rich insights into participants' perceptions, experiences, and observations regarding clothing symbolism of the President's inaugural dress. Examples of questions included inquiries about the religious and sociological significance of Adinkra symbols and the societal implications. The interviews were audio-recorded with participants' consent. These audio recordings were later transcribed into written text for analysis. Detailed notes were taken regarding participants' perspectives, behaviors, and interactions, allowing for a comprehensive insight and analysis. Data processing was conducted using NVivo version 12.1. The analysis followed a thematic analysis approach, aiming to identify recurring themes, patterns, and insights within the interview transcripts. The process involved multiple steps, starting with familiarization with the data through repeated readings of the transcripts. Initial codes were then generated to identify meaningful units of text. These codes were subsequently organized into broader themes related to clothing symbolism and its influence on identity, behavior, and social interactions.

To ensure the rigor and trustworthiness of the study, several measures were taken. Member checking, where participants were provided with summaries of their interviews to validate accuracy, was conducted (Motulsky, 2021). Peer debriefing and consultation with experienced researchers were undertaken to gain external perspectives on data interpretation. The study also aimed for saturation, ensuring that data collection continued until new insights ceased to emerge, enhancing the depth and comprehensiveness of the findings.

### 3.3 Data analysis

The process of deriving and interpreting themes from qualitative data involved a systematic coding process followed by theme consolidation and identification for this study. Interview transcripts and observation notes were initially coded meticulously line-by-line after which descriptive codes were assigned to segments of text relevant to the study's research questions. Recurring patterns and concepts were then identified, which helped in the facilitation of the grouping of similar codes into broader categories or themes. Constant comparison was employed to ensure consistency and accuracy in code application throughout the coding process where subsequent consolidation of related codes into overarching themes and organizing them based on the relevance to the research questions, frequency, consistency, richness, depth, and variability within the dataset was made. Themes were further selected based on their ability to provide detailed insights into the role of clothing symbolism capturing diverse perspectives, experiences, and contexts.

#### 3.3.1 Ethics statement

Prior to data collection, the researchers obtained informed consent from all participants. The consent was voluntary, specific, and offered without coercion, bribery, or misinformation of any kind. This process included explaining the research purpose, the nature of the study, and the rights of the participants. Participants were asked to voluntarily agree to participate and sign consent forms. Confidentiality and anonymity were maintained by using pseudonyms (anonymized) and securely storing audio recordings and transcripts. Respecting the privacy and safety of participants was paramount. The study adhered to ethical guidelines for research. Ethical clearance was obtained from the Takoradi Technical University Ethical Review Committee.

## 4 Results

This section highlights the results of the study based on the key research questions of the study. The key research questions talk about how adinkra symbols on the President's outfit is interpreted religiously, the sociological interpretations, and the societal significance and interpretations of these adinkra symbols.

### 4.1 Research question 1: how are the Adinkra symbols on the president's inaugural outfit interpreted religiously?

Adinkra symbols are rich in spiritual significance in Ghanaian cosmology, often functioning as visual theology through which moral, ethical, and religious ideals are conveyed (Amoateng, 2018). Rather than having a singular or fixed meaning, these symbols invite interpretation based on context, individual belief, and collective cultural memory. For instance, the symbol Sankofa, prominently displayed on the President's attire, was read by several participants as a spiritual invocation. One respondent interpreted its religious dimension as a public call for reflection and renewal:

“*Sankofa teaches us that to move forward, we must return to the lessons of our ancestors. The President wearing this symbol is a declaration that he has learned from past leadership experiences and is prepared to apply divine wisdom in governing the nation.”*

Yet, others offered slightly different readings. One participant emphasized its moral, rather than strictly religious, implication:

“*Sankofa is a reminder that our moral and spiritual growth depends on learning from our past mistakes. The President's outfit symbolized a call for moral renewal and divine guidance in leading the nation.”*

These interpretations align with Anyigor and Olekaibe's (2022) view that African symbols are dynamic theological resources fluid and open to re-interpretation rather than fixed representations. However, they also show subtle variation: while both readings link the symbol to divine wisdom, one centers leadership responsibility, and another emphasizes spiritual correction.

The symbol Gye Nyame, translating as “Except for God,” was similarly understood in deeply spiritual terms. One participant remarked:

“*Gye Nyame reminds us that no ruler, no political party, no human structure is above God. The President's decision to wear it is a spiritual proclamation that his leadership is under divine direction.”*

Another participant went further, describing it as the most powerful religious symbol in the Ghanaian symbolic repertoire:

”*This is the most powerful religious symbol in Ghana. By wearing it, the President communicated that his leadership is ultimately guided by divine will, reinforcing the belief that only God has absolute power over human affairs.”*

While both perspectives affirm the symbol's religious weight, they suggest different emphases, one on divine hierarchy and the other on political humility under divine oversight. Such variations illustrate the symbol's polyvalence.

The Nyame Dua (Tree of God's Altar), also featured on the outfit, was read by participants as a request for divine guardianship and spiritual legitimacy. Its religious resonance, however, was not taken for granted. One participant noted:

“*This symbol reminds us that God's presence must be sought in all things. The President is publicly declaring his dependence on divine guidance for his leadership.”*

Another observed:

“*The President is making a public declaration that he acknowledges God's presence in his leadership. This is not just fashion; it's a spiritual statement that aligns his governance with moral uprightness.”*

These statements point to a shared reverence, but again, the meanings diverge subtly from a theological alignment to a performative spiritual declaration. As Akinbileje (2014) explains, symbols like Nyame Dua are embedded with layers of ritual and political meaning, shaped by the wearer's context and the observers' interpretive frameworks.

Finally, Dwennimmen (Ram's Horn), often associated with humility, sparked reflection about leadership ethics. One participant offered:

“*Even the strongest must bow before God. This symbol is a reminder that leadership should not be arrogant or self-serving but guided by humility and divine wisdom.”*

Yet, another viewed the symbol less as a divine directive and more as a social philosophy:

“*It is a lesson in leadership, true strength lies in humility. The President's outfit sends a message that, despite his power, he remains humble before God and the people.”*

As Mawuli (2019) notes, symbols like Dwennimmen reinforce not only spiritual but also civic values, such as humility, restraint, and ethical leadership. The respondents' comments illustrate this tension and complementarity between divine accountability and public service.

This section demonstrates that while Adinkra symbols carry strong religious associations, their meanings are not uniform or singular. Instead, interpretations are shaped by individual experience, cultural literacy, and spiritual disposition. By foregrounding multiple voices, this study demonstrates how symbols such as Sankofa, Gye Nyame, Nyame Dua, and Dwennimmen serve as contested yet cohesive languages through which the President's moral and religious commitments are publicly interpreted.

### 4.2 Research question 2: what are the sociological interpretations of the Adinkra symbols on the president's inaugural outfit?

From a sociological standpoint, the Adinkra symbols worn by the President during his inauguration were widely interpreted as potent expressions of cultural identity, political legitimacy, and moral leadership. Participants underscored how these symbols function as non-verbal communicative tools deeply embedded within Ghanaian cultural semiotics, capable of conveying nuanced socio-political messages to a diverse public. The use of Adinkra symbols thus exemplifies a performative act of leadership, where dress becomes a form of political discourse grounded in indigenous epistemologies (Aflakpui et al., 2023).

The Sankofa symbol was notably understood as a call for cultural reclamation and introspection. Participants connected the symbol to a perceived moral decline in contemporary Ghanaian society, interpreting its use as a critique of uncritical modernization and a rallying call to embrace indigenous governance ethics. As one participant explained:

“*Sankofa reminds us to return to what worked for us in the past. It speaks to the need for leaders to embrace indigenous wisdom in governance rather than relying solely on Western models.”*

This interpretation aligns with Aflakpui et al. (2023), who view Adinkra symbols as “cultural touchstones” that help societies maintain continuity amid change. Another participant added:

”*We have lost so much of our cultural identity to Western influence. By wearing Sankofa, the President reminds us to reconnect with our roots and preserve our traditions.”*

These statements echo ongoing scholarly debates about cultural authenticity, postcolonial identity, and the politics of dress in African governance. The Nyame Dua, when viewed sociologically, symbolized the moral and spiritual grounding of leadership. Its presence was not merely decorative but interpretively linked to the sacred obligations of political authority. Participants saw the symbol as a reminder that power must be tempered by divine accountability and moral responsibility. One respondent emphasized:

“*This is a powerful message. It suggests that Ghana's governance should be based on ethical leadership, honesty, and service to the people.”*

This analysis complements Akinbileje (2014), who asserts that African visual symbols often serve as moral signposts guiding civic behavior and leadership conduct. Here, the Nyame Dua fosters a model of governance rooted in justice, humility, and collective wellbeing. The Gye Nyame symbol elicited strong emotional and nationalistic interpretations. Participants viewed it as a unifying emblem of resilience and hope amid Ghana's socio-economic challenges. The invocation of divine sovereignty through this symbol was interpreted not as a passive appeal to religion but as an affirmation of the Ghanaian spirit of perseverance and collective destiny. One participant declared:

“*We have seen economic hardship, corruption, and leadership struggles. Gye Nyame reassures us that no matter the challenges, God remains the final authority over our nation's fate.”*

This response supports Amoateng's (2018) argument that Gye Nyame transcends specific religious affiliations to become a universal symbol of collective faith. Moreover, its usage here resonates with Ankora's (2022) findings that Gye Nyame, as part of national emblems, reflects the aspirational ethos of the Ghanaian people. Its inclusion on the President's attire thus communicated a message of endurance, legitimacy, and divine oversight in statecraft.

Lastly, the Dwennimmen (Ram's Horn) symbol was interpreted as a radical reframing of leadership ideals. Rather than endorsing dominance, participants believed it signified the importance of servant leadership, humility, and ethical strength. As one respondent noted:

“*This is a message to those in authority: true strength is not in force or arrogance but in service to the people.”*

Such interpretations challenge masculinist and authoritarian tropes often associated with African leadership, proposing instead a model where humility coexists with resolve. This aligns with Jecty (2022), who suggests that Adinkra symbols function as “ethical blueprints,” reinforcing the importance of accountability and moral responsibility in leadership. Adom et al. (2018) further contextualize Dwennimmen within frameworks of environmental and social sustainability, proposing that symbols like these guide leaders to prioritize long-term communal wellbeing over immediate political gains.

Collectively, these sociological interpretations demonstrate that the Adinkra symbols on the President's outfit were not simply aesthetic choices but constituted a layered, culturally grounded narrative of leadership. Through these symbols, political authority is situated within a broader moral and spiritual framework, enabling citizens to interpret state power through indigenous cultural logics and shared ethical expectations.

### 4.3 Research question 3: what is the societal significance and implications of the Adinkra symbols displayed on the president's inaugural outfit?

The Adinkra symbols featured on the President's inaugural outfit were widely interpreted by participants as a form of strategic cultural communication with broad societal implications. These symbols served not only aesthetic purposes but also conveyed powerful ideological and moral messages to the Ghanaian public. Their deployment at a high-stakes political event such as the presidential inauguration functioned as a visual language articulating value of continuity, ethical leadership, and divine trust qualities that resonate strongly in Ghana's socio-political imagination.

Sankofa, symbolizing the importance of learning from the past, was viewed by participants as a deliberate statement of policy intent. It was interpreted as a metaphor for governance renewal, urging leaders and citizens alike to reflect on previous missteps and realign future national strategies accordingly. As one participant noted:

“*By wearing Sankofa, the President is saying: ‘Let us go back and fix what was neglected.' It is a call for national introspection and a promise of policy continuity.”*

Another echoed:

“*Sankofa is a strategic communication tool. It signals the President's intent to revisit effective policies from the past and correct governance failures.”*

These interpretations resonate with Udechukwu (2019), who emphasizes that Adinkra symbols can function as political semiotics, especially during democratic transitions, acting as vehicles for projecting national values. Similarly, Adom et al. (2018) assert that Adinkra serves as an educative tool, cultivating civic awareness and shaping public discourse on governance.

Nyame Dua, associated with sanctity and divine protection, was perceived as a symbol emphasizing integrity, spiritual accountability, and moral responsibility in leadership. Participants regarded it as a reminder that public office should be treated as a divine trust:

“*This symbol tells us that governance should be sacred. Leaders must see their positions as divine responsibilities, not just political offices. This symbol is a call for integrity in public office. It signifies a commitment to restoring trust in leadership through transparency and accountability”*

This interpretation is consistent with Ansong (2022), who notes that such symbols embed ethical codes within leadership structures, projecting a vision of leadership accountable not only to the people but also to higher spiritual standards.

Gye Nyame, perhaps the most spiritually potent of the symbols used, carried themes of resilience, hope, and divine sovereignty. It was viewed as both a reassurance to the public and a declaration of the President's dependence on divine guidance. One participant remarked:

“*Gye Nyame tells us that no matter the hardships, we must trust in God's plan for Ghana. It's a message of faith and perseverance. Gye Nyame assures us that the President believes in God's guidance to overcome corruption, unemployment, and inflation*”

Another elaborated:

“*The attire worn by Mr. Mahama at his inauguration with the ‘Gye Nyame' depicts the faith of Ghanaians who believe in God's involvement in every aspect of human life. Essentially, the new President was emphasizing the centrality of God in his administration and how ‘Only God' or ‘Except God' can make him succeed in his second life at the presidency.”*

This aligns with Amoateng (2018), who argues that the Gye Nyame symbol bridges the spiritual and political realms, reaffirming national identity through shared religious values. Jecty (2022) further argues that such symbols mobilize faith narratives to inspire collective action during times of economic or political uncertainty.

Finally, Dwennimmen, the ram's horns, was widely seen as embodying a leadership philosophy grounded in humility, restraint, and strength. It was interpreted as a message of contrast distancing the new administration from past leadership styles perceived as arrogant or disconnected from the people. A participant explained:

“*This is a declaration that leadership is about serving, not ruling. The President is reminding us that true power is exercised with wisdom and restraint.”*

This reading supports Mawuli (2019), who describes Adinkra symbols as moral compasses within the public sphere, particularly effective in framing leadership as both authority and service.

Taken together, the participants' interpretations underscore that the Adinkra symbols used in the President's attire were more than traditional motifs they were ideologically loaded, serving as a non-verbal manifesto of leadership values. The symbols created a shared semiotic field where citizens could project expectations, hopes, and criticisms. The study thus suggests that the public reception of political symbolism is shaped not only by visual aesthetics but also by a shared cultural literacy and historical memory, which collectively frame how leadership is perceived in Ghana's postcolonial democratic context.

## 5 Discussions

The religious interpretations of the Adinkra symbols on the President's inaugural outfit reveal a profound intersection between faith, governance, and cultural identity in Ghanaian society. These interpretations align with Symbolic Interactionism Theory, which posits that meaning is not inherent but is socially constructed through interaction (Blumer, cited in Mardon et al., 2021). The President's deliberate selection of these symbols on his attire serves as a communicative act, reflecting shared religious beliefs and reinforcing moral and spiritual narratives within the national consciousness. Additionally, the Semiotic Theory of Fashion supports the argument that clothing is more than a material artifact; it operates as a system of signs that convey deeply embedded societal and theological meanings (Barthes, cited in Karunaratne, 2018).

Fashion operates as a system of signs, and its role in political communication can be deeply understood through the lens of Roland Barthes' Semiotic Theory of Fashion. Barthes (1990) argued that clothing, beyond its functional use, serves as a language a symbolic system through which meanings are encoded and decoded. In political settings, attire is not arbitrary but often a carefully curated symbol of ideology, morality, or cultural affiliation. This is evident across Africa and beyond. For instance, in post-World War II Nigeria, women used specific clothing styles to protest colonial taxation (Allman, 2004), while in Tanzania, state interventions in dress codes sparked public contestation over morality and identity (Allman, 2004). Kenya's adoption of European dress and Zanzibar's regulation of Swahili attire also demonstrate how fashion plays into national identity construction (Allman, 2004). Globally, garments have served as resistance tools from Sudanese women wearing trousers in defiance of conservative laws to European debates over Muslim women's headscarves, each clothing choice becoming a site of ideological negotiation (Behnke, 2016).

These comparative examples demonstrate that fashion in political life often performs strategic, symbolic, and ideological functions. African leaders like Kwame Nkrumah used kente cloth to embody Pan-African pride (Arthur, 2001), In this broader context, the Ghanaian president's attire, often interpreted strictly through a religious lens, may also serve as a calculated semiotic act, signaling humility, cultural rootedness, or solidarity with ordinary citizens. His choices in fabric, color, and silhouette form part of a larger political performance that aligns with global practices of sartorial symbolism. Thus, through deeper semiotic deconstruction and comparative analysis, we can see how fashion communicates political meaning and contributes to the construction of leadership identities in culturally resonant and ideologically charged ways.

Fashion has long served as a powerful medium for political expression. From Melania Trump's pussy-bow blouses and the pink hats worn by participants in the Women's March, to Ivanka Trump's Mandarin-collared dress during her meeting with the Chinese president, clothing in political contexts conveys distinct messages about beliefs, unity, and dissent (Neema Amani, 2025). Designers, whether Alexander McQueen or Raf Simons, have continually used fashion to voice opinions and shape perspectives on global affairs (Neema Amani, 2025). Be it through provocative slogans, deliberate color choices, or symbolic accessories, fashion professionals maintain a complex, often contradictory relationship with politics, one marked by continuous engagement (Neema Amani, 2025). Ultimately, fashion functions as a symbolic language, carrying meanings that transcend surface appearance. The garments and adornments we choose are deeply tied to the societal structures and cultural narratives of our time.

Through wearing these symbols, the President engaged in a symbolic discourse that resonated with the spiritual convictions of the populace, reinforcing leadership as a divine responsibility rather than a mere political role. The presence of the Sankofa symbol on the President's attire was widely interpreted as a call for moral reflection and spiritual renewal. Rooted in the principle of retrieving wisdom from the past to shape the future, it is a powerful religious emblem that signifies repentance and divine reorientation (Anyigor and Olekaibe, 2022). This aligns with symbolic interactionism, which highlights that individuals assign meanings to symbols based on shared societal experiences (Carter and Fuller, 2016). Theologically, Sankofa embodies biblical and indigenous African teachings that emphasize the importance of learning from past errors to attain moral and spiritual progress. This perspective is consistent with Amoateng's (2018) assertion that Adinkra symbols function as “visual theology,” embedding religious tenets into Ghanaian life. The President's use of Sankofa on his attire can thus be viewed as a symbolic sermon, an acknowledgment of past governance mistakes and a commitment to rectify them under divine guidance. The Gye Nyame symbol, widely regarded as the ultimate assertion of divine sovereignty, was another focal point as it was seen as a reaffirmation of faith in God's supreme authority over human affairs, including governance. This aligns with the semiotic theory of fashion, which posits that fashion operates as a communicative tool that conveys ideological and theological values beyond aesthetics (Boero, 2023). By wearing Gye Nyame, the President effectively engaged in a non-verbal proclamation of the divine basis of his leadership, reinforcing the idea that earthly power is subordinate to spiritual authority. This confirms Amoateng (2018), who argues that Adinkra symbols serve as embedded theological messages that guide moral and ethical conduct. This also resonates with Blumer's (cited in Mardon et al., 2021) argument that symbols acquire significance through social interaction; in this case, Gye Nyame is not merely a design on fabric but a shared affirmation of divine governance. Furthermore, the perception of Gye Nyame as a statement on political humility suggests that the President's outfit was not just a visual representation of Ghanaian culture but a deliberate theological declaration of servitude to God. This is particularly significant in a nation where religious faith remains deeply intertwined with political legitimacy. The presence of the Nyame Dua symbol on the President's attire was largely understood as an invocation of divine protection and guidance. This aligns with Akinbileje (2014), who emphasizes that symbols such as Nyame Dua are not merely decorative but serve as spiritual invocations within African textile traditions. From a symbolic interactionist perspective (Stryker and Annual Reviews, 2008), the presence of Nyame Dua reinforces the idea that meaning is negotiated within specific cultural and religious contexts. In Ghana, where divine guidance is considered essential for governance, this symbol serves as a reminder that political authority is ultimately subject to divine scrutiny. Moreover, the religious interpretation of Nyame Dua aligns with Anyigor and Olekaibe's (2022) argument that African religious symbols act as bridges between the material and spiritual worlds. This highlights how political leadership in Ghana is not just a secular role but a spiritual covenant with the people and God. The inclusion of Dwennimmen on the President's attire was widely seen as a reminder that true power must be exercised with humility. The findings reflect the semiotic theory of fashion, which argues that clothing is a medium through which social roles and expectations are communicated (Karunaratne, 2018). In this case, the President's attire was read as an ethical statement, reinforcing the idea that leadership should be exercised with grace and deference. Furthermore, this aligns with Carter and Fuller (2016), who argue that symbols operate as dynamic communicative tools that influence perception and action. By wearing Dwennimmen, the President engaged in a public reaffirmation of his commitment to servant leadership. This is particularly significant given the political landscape, where humility is often contrasted with authoritarianism. The respondents' assertion highlights how Dwennimmen functions as both a moral guide and a leadership ethos, reinforcing the expectation that power must be exercised responsibly.

The sociological interpretations of the Adinkra symbols on the President's inaugural outfit highlight their role as powerful visual narratives that communicate leadership philosophy, national identity, and cultural values. These symbols function as a non-verbal medium through which Ghanaian leadership conveys its vision, legitimacy, and commitment to societal progress. The findings demonstrate how the Adinkra symbols, Sankofa, Nyame Dua, Gye Nyame, and Dwennimmen serve as ideological anchors that reinforce Ghana's sociopolitical ideals and cultural heritage. The presence of the Sankofa symbol on the President's outfit was widely interpreted as a call for cultural revival and the restoration of Ghanaian values. This interpretation aligns with Aflakpui et al. (2023), who argue that Adinkra symbols function as cultural touchstones, ensuring that societal values remain intact even amid modernization. Participants in this study expressed concerns over the erosion of traditional ethics, morality, and leadership standards, attributing this decline to the growing influence of westernization. The Sankofa symbol, therefore, was seen as a deliberate invocation of indigenous wisdom, urging both leaders and citizens to reengage with foundational Ghanaian principles. Symbolic interactionism provides a useful framework for understanding this interpretation. As Blumer (cited in Mardon et al., 2021) suggests, symbols are socially constructed and derive their meaning from interaction. The public's engagement with the Sankofa symbol reflects an ongoing negotiation of national identity, where the past is continually referenced to shape contemporary governance. Moreover, Brooks-Yip (2023) emphasizes that meaning-making through symbols plays a crucial role in identity construction, further reinforcing the idea that the President's adoption of Sankofa is not merely aesthetic but a strategic socio-political statement aimed at fostering cultural continuity. The Nyame Dua symbol was interpreted as a representation of the fusion between governance and spirituality, reinforcing the belief that leadership is a sacred duty requiring moral and ethical integrity. This interpretation is consistent with Akinbileje (2014), who asserts that African symbols serve as ethical signposts guiding leadership and social conduct. The placement of Nyame Dua on the President's attire was perceived as an affirmation of the commitment to just, inclusive, and morally upright leadership. From a semiotic perspective, Barthes (cited in Karunaratne, 2018) argues that clothing functions as a system of signs where garments transcend their material form to communicate deeper ideological messages. In this case, Nyame Dua signals an appeal to moral governance, urging national leaders to prioritize honesty, justice, and service to the people. Participants emphasized that the presence of this symbol was a reminder that governance should not be driven by self-interest or corruption but should be anchored in divine principles. This finding supports Carter and Fuller (2016), who highlight that symbolic interpretations influence social behavior by embedding cultural values into daily practices. By wearing Nyame Dua, the President symbolically aligns his leadership with ethical responsibility, reinforcing public expectations of moral governance. The interpretation of Gye Nyame as a symbol of national resilience and divine sovereignty aligns with Amoateng (2018), who describes the symbol as a sociocultural anchor reinforcing Ghana's faith and endurance in the face of adversity. Participants in this study linked the presence of Gye Nyame on the President's attire to Ghana's ability to navigate economic struggles, political instability, and leadership challenges while maintaining hope in divine providence. Symbolic interactionism provides further insight into this interpretation. Mead's concept of “taking the role of the other” (as cited in Stryker and Annual Reviews, 2008) explains how individuals internalize collective meanings, shaping societal expectations. The widespread recognition of Gye Nyame as a national emblem (Ankora, 2022) illustrates its role in fostering collective resilience. Its presence on the President's outfit, therefore, was perceived not just as a religious symbol but as a unifying motif that reassures citizens of Ghana's strength and continuity. This perspective is also reflected in Husin et al. (2021), who argue that symbols within cultural contexts often serve as mechanisms for collective reassurance and national solidarity. Gye Nyame's placement on the President's attire can thus be seen as a deliberate act of political communication, signaling confidence in Ghana's ability to overcome its challenges through unity and faith.

The Dwennimmen symbol, which embodies both strength and humility, was interpreted as a challenge to conventional notions of authority. Participants noted that this symbol promotes the idea of servant leadership, where power is exercised through service rather than dominance. This aligns with Jecty (2022), who asserts that Adinkra symbols act as ethical blueprints, reinforcing principles of integrity and accountability in leadership. The sociological relevance of this interpretation is significant. As Aflakpui et al. (2023) argue, Adinkra symbols serve as visual indicators that define societal expectations and values. The use of Dwennimmen in the President's attire can thus be understood as an implicit commitment to humility and responsible leadership. This perspective is supported by Adom et al. (2018), who contend that symbols like Dwennimmen encourage long-term societal wellbeing over short-term political gains. The interpretation of this symbol resonates with Mead's (cited in Stryker and Annual Reviews, 2008) emphasis on social interaction as a means of shaping identity. By incorporating Dwennimmen into his attire, the President publicly negotiates his leadership identity, aligning it with values of humility, strength, and service. Furthermore, the semiotic approach highlights how symbolic elements in fashion contribute to leadership representation. Barthes (cited in Karunaratne, 2018) underscores that every component of attire carries meaning within its cultural framework. The inclusion of Dwennimmen thus serves as a counter-narrative to authoritarian leadership styles, reinforcing ideals of ethical governance and collective responsibility.

The societal significance and implications of the Adinkra symbols displayed on the President's inaugural outfit highlight the deep-rooted cultural, ethical, and political messages embedded within Ghanaian symbolism. These symbols serve as tools of communication, governance, and social cohesion, reflecting the expectations of leadership and national direction. They were examined through the interpretations of the Adinkra symbols Sankofa, Nyame Dua, Gye Nyame, and Dwennimmen, using the lens of Symbolic Interactionism and the Semiotic Theory of Fashion, supported by empirical insights from existing literature. The interpretation of Sankofa as a policy-oriented symbol aligns with Symbolic Interactionism, which posits that meanings are socially constructed through interaction and negotiation (Mardon et al., 2021). Respondents associated Sankofa with governance introspection, acknowledging past failures while emphasizing the need for corrective action. This reflects Udechukwu (2019), who asserts that traditional African symbols serve as political statements that influence national consciousness during leadership transitions. Furthermore, the use of Sankofa as a strategic communication tool corresponds with Adom et al. (2018), who argue that Adinkra symbols act as educational frameworks that shape governance and public administration. From a semiotic perspective, the presence of Sankofa on the President's outfit transforms clothing into a communicative device, encoding messages of reform and accountability. Barthes' (as cited in Karunaratne, 2018) conceptualization of fashion as a language of signs is evident here, as Sankofa signals a commitment to revisiting policies that were either abandoned or ineffective. By incorporating this symbol, the President aligns his administration with national aspirations for policy continuity and correction of governance errors, reinforcing his credibility and legitimacy. The interpretation of Nyame Dua as a symbol of ethical governance, accountability, and divine responsibility resonates with the central tenets of symbolic interactionism. As Blumer (cited in Mardon et al., 2021) posits, meanings emerge from social interaction and are constantly negotiated. In this context, the symbol is interpreted as a reminder that governance should be sacred and that leadership is a moral duty rather than a mere political function. This aligns with Ansong (2022), who emphasizes that Adinkra symbols often communicate ethical imperatives in leadership. The assertion that “leaders must see their positions as divine responsibilities” suggests a deliberate social construction of power, where leadership is legitimized through religious and ethical discourse. This reflects Stryker and Annual Reviews (2008) view that the self is formed through interactions that reinforce societal expectations. Here, the President is symbolically negotiating his role as an ethical leader committed to transparency and public trust, an interpretation that underscores the intersection of religion, morality, and governance in Ghanaian political culture. From a semiotic perspective, Nyame Dua's placement on the outfit signifies a performative act of leadership. According to Boero (2023), fashion operates as an axiological system that transmits societal values. The use of Nyame Dua on the President's attire reflects the sacralization of governance, reinforcing the notion that leadership should be based on divine principles and ethical integrity. This interpretation establishes a moral contract between the leader and the governed, echoing the traditional African philosophy of leadership as a duty to the collective wellbeing. The Gye Nyame symbol was interpreted as a message of faith, resilience, and divine reassurance, reflecting the deep spiritual consciousness of Ghanaian society. Participants perceived its presence as an assertion of the President's belief in divine guidance, particularly in tackling national challenges such as corruption, unemployment, and inflation. This supports Amoateng's (2018) argument that religious symbols enhance political credibility by aligning leadership with national spiritual beliefs. From the perspective of symbolic interactionism, Gye Nyame functions as a collective symbol of resilience, reinforcing the idea that leadership and national stability are contingent upon divine will. Mead's (as cited in Stryker and Annual Reviews, 2008) emphasis on the internalization of societal expectations is evident in this interpretation, as the President's use of the symbol reflects public perceptions of faith as a guiding principle in governance. Additionally, the assertion that “Gye Nyame assures us that the President believes in God's guidance” aligns with the semiotic principle that symbols communicate ideological commitments (Inayatullah and Wahjudi, 2023). As Boero (2023) argues, fashion can act as a medium of social values, and in this case, Gye Nyame elevates the political discourse to a spiritual plane, reinforcing the legitimacy of governance through religious symbolism. The presence of Dwennimmen was interpreted as a declaration of leadership through humility, wisdom, and service. This interpretation resonates with Mawuli (2019), who posits that Adinkra symbols function as ethical blueprints for governance. The assertion that “leadership is about serving, not ruling” suggests a symbolic departure from authoritarian leadership styles toward a governance model rooted in humility and collective responsibility. From a symbolic interactionist perspective, Dwennimmen functions as an interactive symbol that negotiates leadership expectations. Carter and Fuller (2016) argue that meanings are shaped by individual experiences within social contexts, and in this case, participants associated the symbol with a moral critique of past administrations. This aligns with Kotarba's (2014) perspective on translational social research, which emphasizes the role of symbols in bridging historical governance patterns with contemporary expectations. The semiotic interpretation of Dwennimmen also aligns with the notion of fashion as a coded system (Karunaratne, 2018). By incorporating this symbol, the President strategically repositions his leadership narrative, reinforcing an image of wisdom, restraint, and servant-leadership. This interpretation is consistent with Barthes' (as cited in Honeycutt, 2021) argument that clothing choices convey complex social messages, shaping public perceptions of authority and credibility.

The interpretations of Sankofa, Nyame Dua, Gye Nyame, and Dwennimmen align with scholarly perspectives on the political, ethical, and spiritual dimensions of Adinkra symbolism. The findings reinforce Udechukwu (2019) and Adom et al. (2018) regarding the political utility of Adinkra symbols, as well as Amoateng (2018) and Mawuli (2019) on their moral and ethical significance. Ultimately, the President's use of Adinkra symbols serves as a performative act of leadership, reinforcing national values of accountability, humility, resilience, and ethical governance. These symbols negotiate power, establish credibility, and construct a leadership narrative that aligns with the cultural and spiritual ethos of the Ghanaian people.

## 6 Conclusions

This study critically examined the sociological, religious, and fashion interpretations of the Adinkra symbols displayed on the 2025 inaugural outfit of Ghana's President. Using Symbolic Interactionism and the Semiotic Theory of Fashion, the research demonstrated how these symbols serve as powerful communicative tools that convey leadership philosophy, cultural identity, and political ideology. The findings revealed that the Adinkra symbols Sankofa, Gye Nyame, Nyame Dua, and Dwennimmen carry significant religious and sociological meanings. Sankofa symbolized governance, introspection and cultural revival, reinforcing the need for leadership to draw on past wisdom. Gye Nyame emphasized divine sovereignty and national resilience, affirming faith in God's guidance during socio-political challenges. Nyame Dua underscored the sacredness of governance, reinforcing ethical leadership, while Dwennimmen highlighted humility as a fundamental trait of responsible leadership. These symbols collectively shaped public perception of leadership, reinforcing cultural values and moral expectations within Ghanaian society.

Beyond aesthetics, the study established that Adinkra symbols function as strategic tools for shaping political narratives, fostering national unity, and reinforcing Ghanaian identity. Their presence in the President's attire highlights the role of fashion as a medium of socio-political communication, where clothing choices transcend personal expression to become embedded with ideological meanings. Through the lens of Symbolic Interactionism, these symbols are interpreted and made meaningful within cultural and social exchanges, while the Semiotic Theory of Fashion frames clothing as a language through which power, identity, and ideology are encoded and interpreted. This dual-theoretical framework enriches our understanding of how traditional symbols maintain contemporary relevance in governance and national identity formation. The study, therefore, contributes to the literature on African semiotics, political symbolism, and cultural heritage, illustrating how material culture, such as presidential fashion, becomes a dynamic platform for ideological expression and cultural continuity.

## 7 Recommendations for policy and practice

In light of the study's conclusions, Ghana's political and leadership establishments must acknowledge the role that Adinkra symbols play in forming cultural identity, national consciousness, and moral leadership. Making cultural symbols a part of government is one of the main policy recommendations. The National Commission on Culture and the Ministry of Chieftaincy and Religious Affairs ought to create official guidelines that support the use of native Ghanaian symbols, especially Adinkra, in official events, official dress, and national branding. In order to reinforce national identity and cultural continuity, this endeavor should be strengthened by a Presidential Decree or a legislative policy requiring the exhibition of important cultural symbols during state events. Education is another important area where policy involvement is needed. The study of Adinkra symbols and their socio-political and semiotic importance ought to be incorporated into the national education curriculum at both the elementary and university levels by the Ghana Education Service (GES) and the National Council for Curriculum and Assessment (NaCCA). Young Ghanaians would gain a greater awareness of their cultural history and its significance to the government as a result of this. Specialized modules that examine the connections between Ghanaian symbols, political leadership, and national identity should be introduced by universities, especially those that provide programs in African Studies, Sociology, Political Science, and Fashion Design. Policies that support ethical governance by referencing traditional Ghanaian philosophical traditions are also required. To encourage responsibility, humility, and wisdom in governance, the National Development Planning Commission (NDPC) and the Institute of Local Government Studies (ILGS) ought to include Adinkra symbols like Sankofa, Gye Nyame, Dwennimmen, and Nyame Dua in leadership development courses. Formal training on the intellectual and ethical meanings of these symbols should be provided to political leaders and public employees to guarantee that government is based on Ghanaian principles rather than outside influences. Parliament ought to think about passing a Cultural Symbolism Protection Act in order to further preserve and advance Ghana's native symbols. The usage of Adinkra symbols in national rituals, historical teaching, and public leadership would be protected, governed, and encouraged by such a law. This law would strengthen the close relationship between cultural identity and moral leadership by guaranteeing that Ghana's rich cultural legacy is not only conserved but also actively incorporated into the country's political system.

Practical steps must be implemented to strengthen the importance of Adinkra symbols in government and societal consciousness, in addition to policy-level actions. Promoting indigenous symbols in public institutions is one such strategy. The Ministry of Foreign Affairs, the Office of the President, and the Ghanaian Parliament should all actively include Adinkra symbols in their official visual communications, such as flags, government seals, and diplomatic clothing. This would act as an ongoing reminder of the cultural values that ought to direct government. To do this, national institutions should work with cultural historians and traditional leaders to make sure that these symbols are interpreted correctly and used responsibly in governance. Prioritizing public education and knowledge on the function of Adinkra symbols in government is also necessary. Ghanaians should be taught about the historical significance and current applicability of these symbols in leadership and governance through national campaigns spearheaded by the National Commission for Civic Education (NCCE). Furthermore, traditional and religious leaders ought to collaborate with civil society organizations and government agencies to spread the moral and ethical teachings included in Adinkra symbols, especially those pertaining to accountability and integrity in leadership. Including Adinkra symbols in state and presidential clothing would be a more realistic strategy. Guidelines for integrating traditional emblems into official state apparel should be developed in cooperation with designers and cultural specialists by the Ministry of Tourism, Arts, and Culture. Additionally, the creation of state clothing that honors Ghanaian tradition should be promoted by the Ghana Fashion Designers Association (GFDA) and regional textile companies. In addition to strengthening cultural diplomacy, this project would guarantee that leadership continues to be symbolically and visually linked to the ideals of the populace.

Incorporating these recommendations into policy and practice, national leadership would continue to be centered on cultural identity, and the significance of traditional Ghanaian symbols in governance would be strengthened. The adoption of Adinkra symbols as moral and ideological markers will be a potent tool for promoting responsibility, national cohesion, and responsible leadership as Ghana continues to negotiate modernization and outside influences.

## 8 Limitations of the study

While this study offers valuable insights into the religious, sociological, and fashion dimensions of Adinkra symbols within Ghanaian political culture, several limitations should be acknowledged.

First, the study focuses exclusively on the 2025 presidential inauguration, which may limit the generalizability of its findings to other political, religious, or socio-cultural contexts where Adinkra symbols are also widely employed, such as funerals, festivals, and commercial branding. This narrow scope restricts a holistic understanding of the broader symbolism of Adinkra in public life.

Second, the use of purposive sampling targeting fashion designers, traditional leaders, museum curators, and cultural preservationists, though methodologically appropriate for qualitative inquiry, may have introduced selection bias. The perspectives captured are valuable but may not reflect the broader Ghanaian public's interpretations of the president's attire.

Third, the modest sample size of 28 participants, while sufficient for in-depth qualitative analysis, limits the representativeness of the findings. The views presented should therefore be interpreted as context-specific rather than universally applicable.

Fourth, the interpretive thematic analysis relied heavily on participant narratives and researcher interpretation. While valid within qualitative paradigms, this approach introduces subjectivity and may omit alternative symbolic readings or underexplored sociopolitical nuances embedded in the Adinkra symbols.

Additionally, the interviews did not consistently use probing techniques to critically assess participants' assumptions especially concerning the authenticity or strategic use of symbolic attire by political figures. This may have led to surface-level interpretations that underplay deeper political performativity.

Lastly, the study did not incorporate complementary sources such as political speeches, media commentary, or public discourse surrounding the inauguration. Such sources could have offered richer contextualization of how the president's symbolic attire was received, interpreted, or contested in the public sphere.

Despite these limitations, the study makes a significant contribution to understanding Adinkra as a powerful medium of cultural expression and political messaging. Future research should broaden the analytical scope by including public perspectives, media discourse, and other political or cultural settings where Adinkra symbols are actively used.

## 9 Positionality statements and novice researchers

Positionality is integral to the practice of qualitative research and demands that researchers, especially novice or postgraduate researchers, engage in deep reflexivity to examine how their identities, values, experiences, and relationships influence all phases of the research process. As Holmes (2020) explains, novice researchers may initially struggle to articulate their positionality concerning their research, but doing so is essential for cultivating ethical and reflexive scholarship. Researchers occupy shifting positions as insiders or outsiders, which can bring both advantages and limitations to data collection and interpretation. Structured learning environments, such as the Racism Untaught workshops, demonstrate how onboarding activities can serve as important entry points for fostering self-awareness around issues of race, bias, and identity. These workshops have shown that understanding one's positionality is a life-long process that contributes to anti-oppressive and culturally responsive design practices (Moses and Elzey Mercer, 2022). This reflective practice is especially critical in disciplines like graphic design, where Western ideologies often dominate educational models in postcolonial contexts such as Ghana. Mensah Bonsu et al. (2025) argue that without incorporating indigenous Ghanaian artforms and philosophies into graphic design education, students are limited in their ability to form authentic cultural design identities. For novice researchers and student designers alike, engaging with their positionality is a necessary step toward decolonising curricula, producing culturally grounded work, and contributing to inclusive, globally relevant creative fields.

## Data Availability

The raw data supporting the conclusions of this article will be made available by the authors, without undue reservation.
